# The effect of disulfidptosis induced by glucose starvation in SLC7A11^high^ ovarian cancer cells

**DOI:** 10.3389/fcell.2025.1608150

**Published:** 2025-12-08

**Authors:** Yating Chen, Yu-Hua Ou, Wenjing Shi

**Affiliations:** Department of Gynecology, The Second Affiliated Hospital of Guangzhou Medical University, Guangzhou Medical University, Guangzhou, China

**Keywords:** ovarian cancer, disulfidptosis, SLC7A11, glucose starvation, disulfide bond

## Abstract

**Objective:**

Disulfidptosis is a glucose deprivation-induced cell death mechanism driven by disulfide stress-mediated actin cytoskeleton disintegration. This study explored whether SLC7A11^high^ ovarian cancer (OC) undergoes disulfidptosis under conditions of glucose starvation. This study aimed to provide a new treatment option for OC.

**Methods:**

Initially, we carried out an assay to assess the cell viability of IOSE 80 and OVCAR 3 cells under conditions of glucose sufficiency and starvation. Subsequently, we examined the difference in SLC7A11 expression between the two cell types under the specified conditions. The intracellular NADP^+^/nicotinamide adenine dinucleotide phosphate (NADPH) ratio and the oxidized glutathione disulfide/reduced glutathione (GSSG/GSH) ratio were quantified using the test kit. In addition, the effect of SLC7A11 on cell death was studied using SLC7A11 transporter activity inhibitors. Cell Counting Kit-8 (CCK-8) was used to investigate the effect of intracellular disulfide bond levels on cell death due to glucose starvation. Finally, we investigated the effect of reactive oxygen species (ROS) on cell death.

**Results:**

The results revealed that the SLC7A11 expression level in OVCAR 3 cells was higher than that in IOSE 80 cells, and OVCAR 3 cells exhibited a lower tolerance to glucose starvation. Glucose starvation stimulates SLC7A11 expression in OVCAR 3 cells, and SLC7A11 facilitates cell death in OC cells under conditions of glucose starvation. Elevated disulfide bond levels in ovarian cancer cells promote cell death under glucose starvation conditions.

**Conclusion:**

Glucose starvation induces high SLC7A11 expression in OC cells, resulting in cell death. This type of cell death is associated with NADPH depletion in the cell and the accumulation of disulfides. Therefore, it is considered that disulfidptosis may occur in SLC7A11^high^ OC cells under glucose starvation conditions.

## Introduction

Ovarian cancer (OC) is the leading cause of death among gynecological cancers, primarily due to the absence of effective early screening and diagnosis methods, resulting in late-stage presentation, high recurrence rates, and significant challenges in diagnosis and treatment. Globally, ovarian cancer ranks eighth in incidence among women, accounting for approximately 3.7% of new cancer cases and 4.7% of cancer deaths in 2020 (Sung et al., 2021). According to a paper from the National Institutes of Health (NIH), the mortality rate of ovarian cancer has decreased compared to that in previous years, but the mortality rate of advanced serous ovarian cancer remains relatively high (Siegel et al., 2024). In the present and the future, the mainstream tumor treatment methods mainly include surgery, radiation therapy, chemotherapy, allogeneic hematopoietic stem cell transplantation, pharmacological hormone therapy, and immune checkpoint inhibitors (Sonkin et al., 2024). Currently, surgery combined with platinum and paclitaxel chemotherapy is the mainstream treatment for OC (Kuroki and Guntupalli, 2020; Wang et al., 2021). Despite the recent significant advancement in surgical and chemotherapeutic treatments for OC, the 5-year survival rate for patients with advanced stages of OC remains low (Baert et al., 2021). Most patients with OC are already at an advanced stage at diagnosis, and some patients with OC develop chemotherapy resistance after treatment (Chiappa et al., 2021; Mirza et al., 2020). The high mortality rate in patients with OC can be ascribed to factors including chemotherapy resistance and extensive intraperitoneal metastasis (Liu et al., 2015; Yang et al., 2018). Therefore, it is essential to identify new effective treatments for OC.

Regulated cell death (RCD) is a form of cell death controlled by a specific molecular pathway, which can be modulated through genetic manipulations or pharmacological interventions (Galluzzi et al., 2018). RCD is integral to body development and is crucial for cellular function in a steady state; it also has a causative association with various diseases, including cancer. The evasion of cancer cell death is considered a hallmark of malignancy, prompting interest in the investigation of alternative RCD mechanisms. For instance, ferroptosis, a type of RCD induced by iron-dependent lipid peroxidation, exhibits distinct morphological and mechanistic features that are different from those of apoptosis (Dixon et al., 2012). Recent research indicates that some cancers resistant to conventional therapy are particularly susceptible to ferroptosis induction (Jiang et al., 2021).

Koppula et al. (2021) demonstrated that cysteine intake facilitated by solute carrier family 7 member 11, also known as xCT (SLC7A11), plays a key role in enhancing glutathione biosynthesis and mitigating oxidative stress and ferroptosis. In 2017, Koppula et al. (2017) reported that SLC7A11 can significantly promote cell death in glucose starvation conditions (Shin et al., 2017; Koppula et al., 2017; Goji et al., 2017). Recently, Xiaoguang Liu et al. reported that SLC7A11 was highly expressed in cancer cells, including UMRC6, H460, A549, and 786-O. The SLC7A11-mediated uptake of cystine for its reduction to cysteine is significantly dependent on the pentose phosphate pathway (PPP), which produces reduced nicotinamide adenine dinucleotide phosphate (NADPH). This elucidates the mechanism by which insufficient NADPH supply hampers the conversion of cystine into cysteine, resulting in excessive NADPH consumption. This process induces actin, a cytoskeletal protein, to form disulfide bond crosslinking and causes cytoskeleton contraction, leading to cell death. This mechanism differs from the previously established forms of cell death and is referred to as disulfidptosis (Liu et al., 2023; Shin et al., 2017; Liu et al., 2020).

SLC7A11 is a cystine–glutamate antiporter that imports one molecule of cystine while exporting one molecule of glutamate (Dixon et al., 2012). Most cancer cells absorb extracellular cystine primarily through the cystine transporter (comprising the catalytic subunit SLC7A11 and the chaperone subunit SLC3A2); cystine is reduced to cysteine for cell utilization (Stipanuk, 2004). Cysteine exists in a reduced state that is unstable *in vitro* and readily oxidizes to form cystine, which contains disulfide bonds (Stipanuk, 2004). Cysteine is an essential amino acid. Cysteine, glutamic acid, and glycine can synthesize glutathione (GSH) by enzymatic activity, which affects cell REDOX homeostasis. Glutathione comprises reduced glutathione (GSH) and oxidized glutathione (GSSG). GSH is a source of thiol in most living cells, playing a crucial function in maintaining proteins in their normal REDOX state. It is a fundamental antioxidant in animal cells (Nguyen and Do, 2022) and is also pivotal in protecting cells from anticancer medicines (Traverso et al., 2013). GSSG is formed by the dehydrogenation of two GSH molecules, resulting in the formation of a disulfide bond.

Previous studies have demonstrated that SLC7A11 expression is significantly elevated in OC cells (Jin et al., 2022; Hong et al., 2021; Fantone et al., 2024). Additionally, Ke et al. reported that SLC7A11 can lead to drug resistance and reduced survival rate of OC by inhibiting cell autophagy (Ke et al., 2021). Therefore, high SLC7A11 expression was reported to be an independent prognostic risk factor for overall survival in OC (Yin et al., 2019). Ma et al. (2019) reported that a low-glucose environment can induce apoptosis in OC cells through mitochondrial and endoplasmic reticulum stress-related pathways, which is also associated with the enhancement of glucose metabolism and OC drug resistance (Sun et al., 2020). Glucose-6-phosphate dehydrogenase in the PPP of glucose metabolism is expected to become a new target for OC treatment (Wang et al., 2023).

This study aimed to explore whether OC undergoes disulfidptosis under glucose starvation conditions. Therefore, we used OVCAR 3 and IOSE 80 cells to demonstrate that ovarian cancer cells have a low tolerance to glucose starvation. Moreover, the cell death of OVCAR 3 cells under glucose starvation conditions was associated with the high expression of SLC7A11 and the increased level of disulfide bonds.

## Materials and methods

### Cell lines and cell culture

OVCAR 3, the human ovarian adenocarcinoma cell line, was utilized in this study and was obtained from the American Type Culture Collection (ATCC, Manassas, VA, United States). IOSE 80, the human normal ovarian cell line, was obtained from the BioVector NTCC Collection Center. All cells were free from *mycoplasma* contamination. The cells were cultured in RPMI-1640 medium supplemented with 10% fetal bovine serum and 100 U/mL penicillin–streptomycin. All cells were incubated at 37 °C with 5% CO_2_.

### Cell viability assay

Cell viability was analyzed using the Cell Counting Kit-8 (CCK-8; Beyotime Biotechnology) according to the manufacturer’s instructions and as described previously (Gao et al., 2019; Gao et al., 2022; Wu et al., 2017). Cells were inoculated into 96-well plates at a density of 1 × 10^4^/well and cultured overnight. Cells were subsequently treated with varying concentrations of glucose for 24 h. Subsequently, 10 µL of CCK-8 was added to each well. Cells were cultured in the dark at 37 °C for 2 h. The D450 value was assessed using a BioTek Synergy HTX Microplate Reader (Agilent, United States), and the results were expressed as a percentage of 100% of the control set.

### Quantitative PCR

Quantitative PCR (qPCR) was performed as previously described (Wu et al., 2017; Zhao et al., 2020). In brief, total RNA was extracted using a tissue/cell RNA rapid extraction kit (Beyotime Biotechnology) according to the manufacturer’s instructions. RNA was reverse-transcribed into cDNA using the PrimeScript™ RT Master Mix (Perfect Real Time) (TaKaRa) according to the manufacturer’s instructions. These cDNAs served as the templates for qPCR. Furthermore, qPCR was performed on the QuantStudio™ 5 (Thermo Fisher Scientific) using TB Green Premix Ex Taq II (Tli RNaseH Plus) (Code no. RR820A). Supplementary Table S1 presents the list of the primers used. The PCR cycling conditions consisted of an initial 30 s denaturation at 95 °C, followed by 40 cycles of denaturation for 5 sec at 95 °C and annealing/elongation for 34 sec.

### Western blot analysis

Western blotting was conducted as previously described (Liu et al., 2025; Ou et al., 2024). Radioimmunoprecipitation assay buffer was administered to OVCAR 3 and IOSE 80 cells to facilitate protein extraction. The extracted protein was used to determine its concentration using an enhanced BCA protein assay kit (Beyotime Biotechnology). Proteins were separated by SDS electrophoresis and transferred onto PVDF membranes. PVDF membranes were blocked with blocking buffer (Beyotime Biotechnology) in buffer for 60 min at room temperature and incubated overnight at 4 °C with primary antibodies. After washing with PBS three times, horseradish peroxidase-conjugated secondary antibody was added and incubated for 1 h at room temperature. Membranes were removed from boxes with blunt forceps after washing with PBS at least three times. One milliliter of BeyoECL Plus (BioSharp) working solution was added to each membrane per 10 cm^2^. Bound antibody was visualized using an enhanced chemiluminescence system (Bio-Rad). The primary antibodies were rabbit polyclonal antibody against beta-actin (Affinity) and rabbit polyclonal antibody against xCT (Affinity).

### Reactive oxygen species assays

The cells were seeded into 6-well plates at 1 × 10^5^/well. Fluorescein diacetate was used together with 10 µM DCFH-DA (2′, 7′-dichlorofluorescein diacetate; Molecular Probes; Beyotime Biotechnology) to label OVCAR 3 and IOSE 80 cells. The cells were incubated in the dark at 37 °C for 30 min, then washed twice with serum-free media and, stored in 1 mL of medium. Intracellular ROS production was quantified using the oxidation of DCFH-DA to fluorescent DCF (2′,7′-dichlorofluorescein). The fluorescence intensity of the treated cells was measured using a fluorescence microscope. The excitation spectrum was 488 nm, while the broad emission spectrum was 525 nm.

### NADP^+^/NADPH detection

Under different experimental conditions, the cells were cultured in a 6-cm cell culture dish for 24 h. After the medium was removed, 400 µL of NADP^+^/NADPH extract was added to each well, and cell lysis was facilitated by aspiration using a pipette. Subsequently, according to the manufacturer’s instructions (Beyotime Biotechnology), samples were used as enzyme standards to measure absorbance at 450 nm. The total NADP^+^ and NADPH concentrations and NADPH concentrations in the cell samples were calculated according to the standard curve.

### GSSG/GSH detection

Cells were manipulated according to the GSSG/GSH assay kit (Beyotime Biotechnology) instructions. The absorbance of the samples at 412 nm was quantified, and a standard curve was drawn to calculate the GSSG/GSH in the cellular samples.

### Drug treatments

First, the cells were inoculated into appropriate culture containers, and the cell concentration was cultivated to 80%. Subsequently, the cell culture medium was replaced with different conditions to observe the effects of various drugs on cell activity.

Sulfasalazine (SAS) is an inhibitor of SLC7A11 transporter activity. We established the high-glucose group (Glu^+^, 25 mM), low-glucose group (Glu^−^, 1 mM), and low-glucose (1 mM) + SAS (1 mM) group (Glu^−^ + SAS). After culturing cells in a cell culture incubator for 24 h, the cell survival rate was detected.

Diethyl maleate can diminish GSH levels in exposed cells and facilitate intracellular disulfide bond formation. Tris(2-carboxyethyl)phosphine (TCEP) can accelerate the S–S reduction reaction, thus reducing disulfide bond formation in the cell. To investigate the promotion or protective effect of drugs on this mode of death, we determined the appropriate treatment duration to achieve a 50% cell survival rate. We found that the cell survival rate was approximately 50% after 12 h of low-glucose treatment. Therefore, we treated the cells with diethyl maleate (1 mM) and TCEP (1 mM) for 12 h, respectively, and then detected the survival rates of the cells in each group.

### Statistical analysis

GraphPad Prism software (version 8.2.2) was used for statistical analyses. Data are presented as the mean ± standard error of the mean (n = 3). Comparisons were carried out using a two-tailed unpaired Student’s t-test. A *p <* 0.05 was considered statistically significant, with *p < 0.05, **p < 0.01, and ***p < 0.001 indicating different levels of significance. ImageJ software and Adobe Photoshop 2021 were utilized for image analysis.

## Results

### OC cells are less tolerant to glucose starvation

**FIGURE 1:**
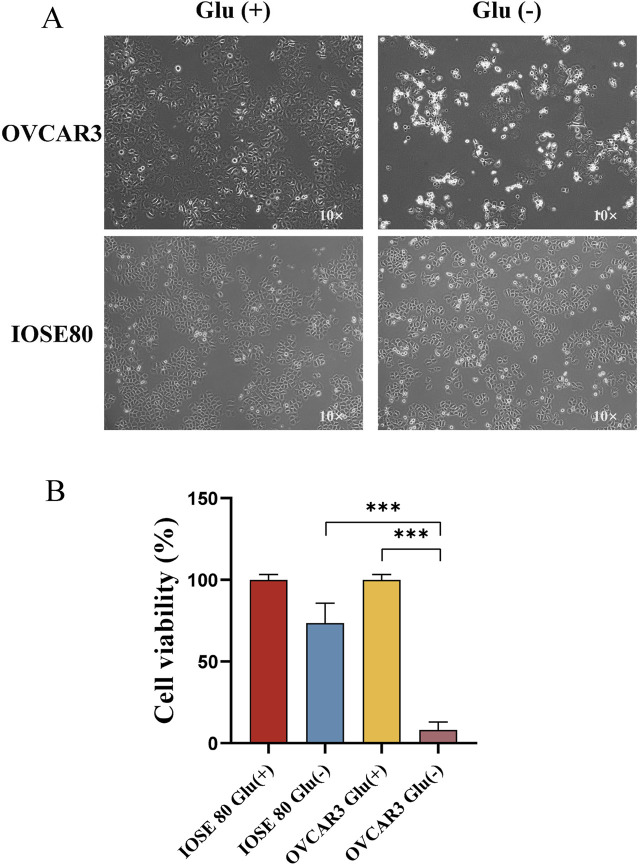
Effect of glucose starvation on IOSE 80 and OVCAR 3 cells. **(A)** Variations in cellular morphology between IOSE 80 and OVCAR 3 cells with or without sugar. **(B)** Effect of glucose starvation on the viability of IOSE 80 and OVCAR 3 cells.

To investigate the tolerance of OC cells and normal ovarian cells to glucose starvation, OVCAR 3 and IOSE 80 cells were exposed to high-glucose medium (25 mM) and low-glucose medium (1 mM) for 24 h, respectively. Figure 1A depicts that the OVCAR 3 cells were atrophic, rounded, and translucent, whereas IOSE 80 exhibited no significant morphological changes. The cell viability of the two cell lines was assessed using the CCK-8 assay. The result revealed that after 24 h of culture in the diet medium, the OVCAR 3 cell survival rate was 8.00% ± 2.85% (mean ± SEM), and the IOSE 80 cell survival rate was 73.47% ± 7.03% (mean ± SEM, *p* < 0.05) (Figure 1B).

### Glucose starvation enhances SLC7A11 expression in OC cells

**FIGURE 2:**
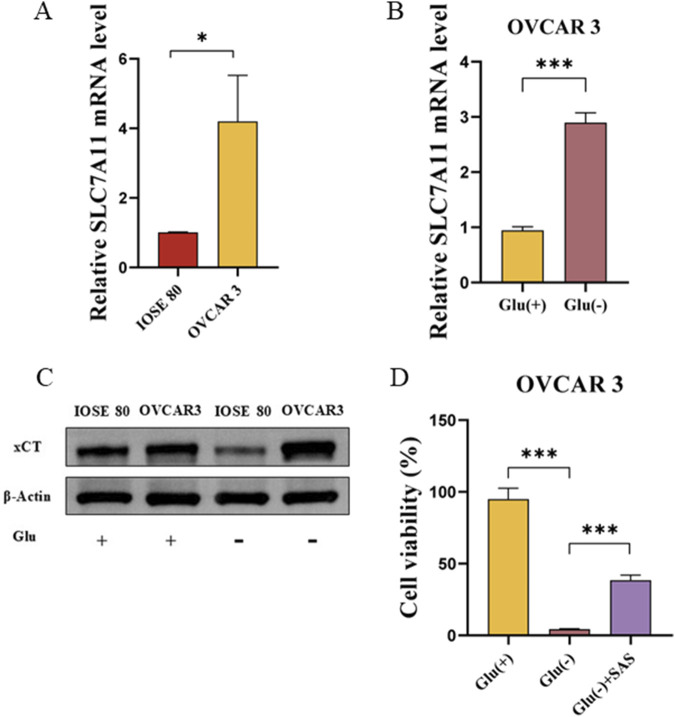
SLC7A11 affects OVCAR 3 cells’ glucose starvation sensitivity. **(A)** SLC7A11 mRNA expression was differentially expressed in IOSE 80 and OVCAR 3 cells. **(B)** Effect of glucose starvation on SLC7A11 mRNA levels in OVCAR 3 cells. **(C)** Effect of glucose starvation on SLC7A11 protein levels in OVCAR 3 and IOSE 80 cells. **(D)** Effect of SAS on glucose starvation-induced OVCAR 3 cell death.

We extracted the mRNA and protein of OVCAR 3 and IOSE 80 cells to understand the difference in SLC7A11 expression between OC cells and normal ovarian epithelial cells. The results revealed that the SLC7A11 expression level was significantly higher in OVCAR 3 cells than in IOSE 80 cells (*p* < 0.05) cells (Figure 2A). However, at the protein level, the relative expression level of SLC7A11 was higher in OVCAR 3 cells than in IOSE 80 cells (Figure 2C).

To investigate the effect of glucose starvation on SLC7A11 expression in OC cells and normal ovarian epithelial cells, we initially examined the differential expression of SLC7A11 in IOSE 80 and OVCAR 3 cells before and after glucose starvation. Figure 2A illustrates that SLC7A11 mRNA expression was increased in OVCAR 3 cells after glucose starvation, and the difference was statistically significant (*p <* 0.05). Western blot confirmed that SLC7A11 protein expression was significantly increased after glucose starvation (Figure 2B).

Sulfasalazine (SAS), an inhibitor of SLC7A11 transporter activity, can impede the xCT absorption of cystine (Pardieu et al., 2022; Liu et al., 2024). To understand whether glucose starvation-induced OC cell death was associated with high SLC7A11 expression, we established the high-glucose group (Glu^+^, 25 mM), low-glucose group (Glu^−^, 1 mM), and low-glucose (1 mM) + SAS (1 mM) group (Glu^−^ + SAS). The cell viability of Glu^+^, Glu^−^, and Glu^−^ + SAS groups were 94.95% ± 4.37%, 4.34% ± 0.23%, and 38.39% ± 2.13%, respectively (Figure 2D). The cell survival rate of the Glu^−^ + SAS group was significantly higher than that of the low-glucose group, with a statistically significant difference (*p <* 0.05).

### In OC cells, glucose starvation induced NADPH depletion and disulfide stress

**FIGURE 3:**
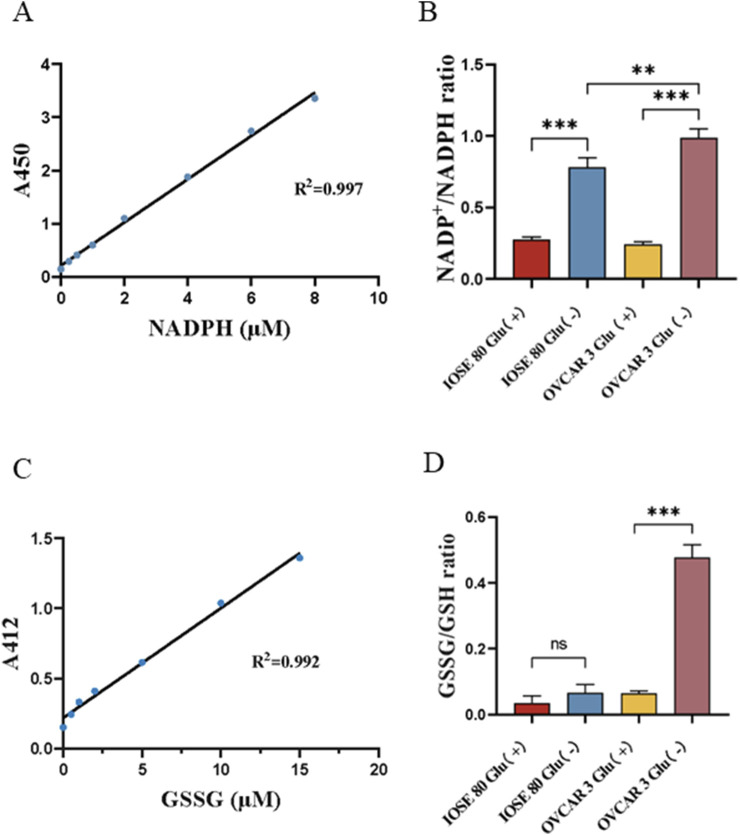
Effect of glucose starvation on the NADP^+^/NADPH ratio and the GSSG/GSH ratio in OVCAR 3 cells. **(A)** Standard curve of NADPH concentration. **(B)** Effect of glucose starvation on the NADP^+^/NADPH ratio in OVCAR 3 cells. **(C)** Standard curve of GSSG concentration. **(D)** Effect of glucose starvation on the GSSG/GSH ratio in OVCAR 3 cells.

Disulfidptosis is characterized by intracellular NADPH depletion and disulfide stress. Therefore, we examined the variations in the NADP^+^/NADPH and GSSG/GSH ratios across different glucose concentrations. The NADP^+^/NADPH ratios of OVCAR 3 cells at varying glucose concentrations were 0.241 ± 0.011 and 0.978 ± 0.036 (*p <* 0.05), respectively (Figures 3A,B). The NADP^+^/NADPH ratios of IOSE 80 cells at different glucose concentrations were 0.274 ± 0.012 and 0.782 ± 0.037 (*p <* 0.05), respectively. However, the GSSG/GSH ratios of OVCAR 3 cells at different glucose concentrations were 0.065 ± 0.004 and 0.477 ± 0.023 (*p <* 0.05), respectively (Figures 3C,D). The GSSG/GSH ratios of IOSE 80 cells at different glucose concentrations were 0.035 ± 0.012 and 0.067 ± 0.015 (*p >* 0.05), respectively.

### Disulfide stress will induce the death of SLC7A11^high^ OC cells caused by glucose starvation

**FIGURE 4:**
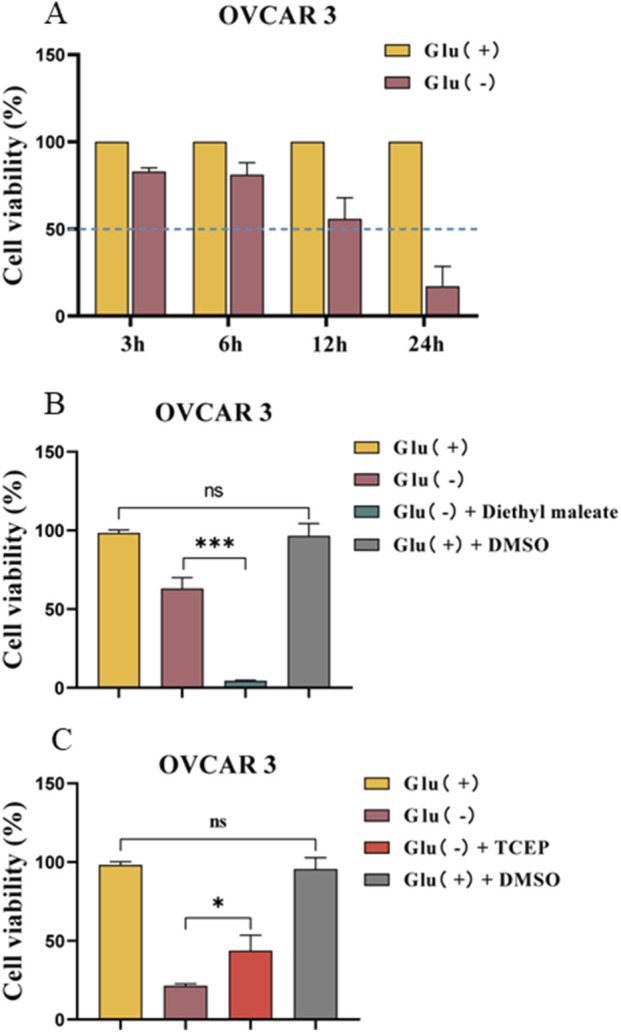
Effect of the disulfide bond level on the mode of death induced by glucose starvation in OVCAR 3 cells. **(A)** Differences in cell viability between OVCAR 3 cells cultured under glucose starvation conditions for different times. **(B)** Effect of diethyl maleate (1 mM) on glucose starvation-induced cell death in OVCAR 3 cells. **(C)** Effect of TCEP (1 mM) on glucose starvation-induced death in OVCAR 3 cells.

To investigate the promotion or protective effect of drugs on this mode of death, we determined the appropriate treatment duration to achieve a 50% cell survival rate. Figure 4A depicts that the cell survival rate was approximately 50% after 12 h of low-glucose treatment.

Diethyl maleate is a thiol-reactive α- and β-unsaturated carbonyl compound that can diminish the GSH levels in exposed cells and facilitate intracellular disulfide bond formation. TCEP can accelerate the S–S reduction reaction, thus reducing disulfide bond formation in the cell. The medium was used to combine the thiol antioxidant maleic acid diethyl ester (diethyl maleate) with the mercaptan reductant TCEP to investigate disulfide bond formation in OC cell death triggered by the glucose starvation mode. The results revealed that the cell survival rate of the Glu^−^ + diethyl maleate group was 4.38% ± 0.24% (Figure 4B). The cell survival rate of the Glu^−^ + TCEP group was significantly higher than that of the low-glucose group (*p <* 0.05) (Figure 4C). However, the control DMSO group exhibited no effect on cell viability.

The formation of intracellular disulfide bonds can induce the death of OC cells exhibiting high SLC7A11 expression under glucose starvation conditions.

### Effects of ROS on OVCAR 3 cell death

**FIGURE 5:**
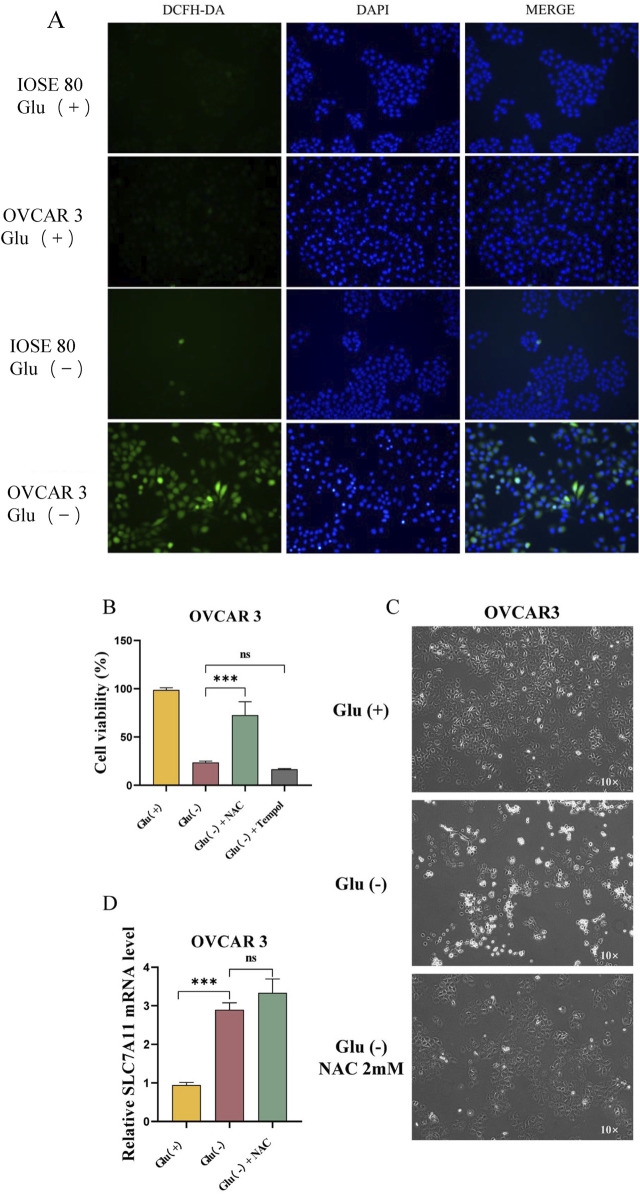
Changes in the ROS levels in OVCAR 3 cells under the glucose starvation condition and the effects of the ROS levels on OVCAR 3 cells. **(A)** Effect of glucose starvation on intracellular ROS levels in IOSE 80 and OVCAR 3 cells. **(B)** Effect of ROS inhibitors Tempol (100 µM) and NAC (2 mM) on glucose starvation-induced death in OVCAR 3 cells. **(C)** Morphological changes between the high-glucose group, the low-glucose group, and the low glucose + NAC 2 mM group. **(D)** Effect of NAC on SLC7A11 expression in OVCAR 3 cells.

To examine the effect of glucose starvation on intracellular ROS levels, we quantified the ROS levels of OVCAR 3 and IOSE 80 cells cultured in high-glucose and low-glucose environments for 24 h. The results revealed that the ROS levels in OVCAR 3 cells were significantly increased under low-glucose conditions (Figure 5A). With the ROS scavengers Tempol and N-acetylcysteine (NAC), the cell survival rates of the Glu^+^, Glu^−^, Glu^−^ + NAC, and Glu^−^ + Tempol groups were 98.71% ± 1.29%, 23.53% ± 0.85%, 72.50% ± 8.10%, and 16.58% ± 0.32%, respectively (Figure 5B). The proliferation of cells in the Glu^−^ + NAC group was gradual, and no cell shrinkage, roundness, or brightening was observed (Figure 5C). No significant difference was observed between the SLC7A11 expression level in OVCAR 3 cells after NAC treatment and the SLC7A11 expression level in the Glu^−^ group (Figure 5D).

## Discussion

OC is one of the most formidable malignancies of the female reproductive system. Globally, ovarian cancer ranks eighth in incidence among women, accounting for approximately 3.7% of new cancer cases and 4.7% of cancer deaths in 2020 (Sung et al., 2021). Although it ranks third in annual incidence among gynecological tumors, it accounts for the highest mortality rate among gynecologic malignancies (Gardner and Chi, 2021). OC frequently exhibits no obvious clinical symptoms in its early stage, with approximately 70% of patients presenting with symptoms including abdominal distension, ascites, and abdominal pain (Kosaka et al., 2021). Consequently, >75% of patients with OC are diagnosed as being in the advanced stage at their initial or definitive diagnosis (Torre et al., 2018). The 5-year survival rate after OC diagnosis remains 47% (Zeng et al., 2022). The prognosis of OC depends on early diagnosis, prompt surgical intervention, and systemic treatment. Surgery combined with platinum-based and paclitaxel-based chemotherapy is the mainstream treatment for OC. Despite advancements in surgical techniques and chemotherapy applications enhancing patients’ prognosis, some patients still develop chemotherapy resistance (Kuroki and Guntupalli, 2020). Significant advancement has been achieved in targeted therapy and immunotherapy for OC; however, more effective OC treatments are required to enhance the prognosis and survival rate of patients with OC.

SLC7A11 is frequently overexpressed in numerous human cancers, and several studies have demonstrated that SLC7A11 overexpression is directly related to the onset and progression of different types of cancer (Ren and Shen, 2019). SLC7A11 is frequently overexpressed in OC (Hong et al., 2021; Fantone et al., 2024; Zhang et al., 2023), and studies have demonstrated that SLC7A11 exerts oncogenic effects by inhibiting cell autophagy and ferroptosis; for instance, Ke et al. reported that SLC7A11 can inhibit cell autophagy, resulting in OC resistance and decreased survival rate (Ke et al., 2021). Hong et al. (2021) reported that PARP inhibition downregulated the cysteine transporter SLC7A11 expression in OC in a p53-dependent manner, leading to reduced GSH biosynthesis, increased lipid peroxidation, and ferroptosis. These studies demonstrate that SLC7A11 expression is highly significant in cell death in OC.

Disulfidptosis necessitates high expression of SLC7A11 and glucose starvation (Liu et al., 2023). We initially cultured OVCAR 3 and IOSE 80 cells and verified that SLC7A11 expression in OC cells was significantly higher than that in normal ovarian cells using qRT-PCR and WB. Subsequently, we changed the glucose concentration in the culture medium, establishing a high-glucose group (25 mM) and a low-glucose group (1 mM) and cultured OVCAR 3 and IOSE 80 cells for 24 h. Initial observation under the electron microscope revealed that under the same low-glucose environment and duration, OVCAR 3 cells exhibited cell shrinkage, rounding, and transparency, while IOSE 80 cells exhibited slower proliferation without any obvious changes in cell morphology. Our research group further used the CCK-8 method to detect cell viability under two conditions, and the results revealed that the OVCAR 3 cell survival rate under low-glucose conditions was significantly lower than that of IOSE 80 cells under the same conditions after 24 h of treatment. The results demonstrated that OC cells have low tolerance to glucose starvation. To examine the correlation between the death mechanism and SLC7A11 expression, we assessed the expression levels of SLC7A11 in OVCAR 3 cells before and after glucose starvation and found that SLC7A11 expression in OC cells was significantly elevated during the glucose starvation condition. These initial findings indicate that the cell death mechanism in OC cells under glucose starvation conditions is related to SLC7A11; however, whether SLC7A11 has a promoting or inhibitory effect remains unknown. Consequently, we added the SLC7A11 active inhibitor SAS to the low-glucose medium to examine the effect of SLC7A11 on this type of cell death. The results revealed that SAS exhibited a protective effect on this type of cell death. Therefore, we concluded that the cell death mechanism in OC cells under glucose starvation conditions is associated with high SLC7A11 expression and that SLC7A11 has a positive effect. Subsequently, we examined whether the cell death mechanism associated with SLC7A11 during glucose starvation conditions is ferroptosis.

The hallmark of double sulfur death is the inadequate availability of NADPH within the cell, which impedes the reduction of cysteine to cysteine, causing intracellular disulfide stress (Liu et al., 2023). GSSG is formed by the dehydrogenation of two GSH through the disulfide bond. Since the number of cells will change after glucose starvation treatment, we measured the reduction in the NADP^+^/NADPH ratio and the GSSG/GSH ratio to mitigate errors arising from cell number differences. We cultured OVCAR 3 and IOSE 80 cells in high- and low-sugar media for 24 h, respectively, and used the NADP^+^/NADPH and GSSG/GSH assay kits to detect the differences in the NADP^+^/NADPH and GSSG/GSH ratios of OVCAR 3 and IOSE 80 cells before and after glucose starvation. The results revealed that the NADP^+^/NADPH ratio of OVCAR 3 and IOSE 80 cells in the low-sugar environment was significantly increased. In glucose starvation conditions, the lack of substrates for the PPP results in a significant reduction in NADPH levels in OC cells and normal ovarian epithelial cells. Combined with the results of our previous experiments, normal ovarian epithelial cells exhibit impaired sugar metabolism due to glucose deficiency, affecting cell proliferation; nevertheless, glucose starvation does not lead to NADPH depletion or cell death. However, this NADPH depletion causes cell death in OC cells. Unlike the change in the ratio of NADP^+^/NADPH, the ratio of GSSG/GSH exhibited a significant difference before and after glucose starvation in OVCAR 3 and IOSE 80 cells. The results revealed that the ratio of GSSG/GSH in OVCAR 3 cells increased significantly after glucose starvation for 24 h, leading to enhanced disulfide bond formation within the cells, while the GSSG/GSH ratio in IOSE 80 cells was not significantly different after glucose starvation for 24 h. As a result, NADPH depletion and disulfide bond formation occurred in SLC7A11^high^ OC cells under glucose starvation conditions. We initially hypothesized that SLC7A11^high^ OC cells’ expression in glucose-starved conditions may undergo dithiol death.

To further investigate whether the cell death mechanism in SLC7A11^high^ OC cells under glucose starvation conditions is associated with disulfide bond formation within the cells, we utilized diethyl maleate, a sulfhydryl-reactive α,β-unsaturated carbonyl compound, to facilitate disulfide bond formation, and TCEP, which accelerates the S–S reduction reaction, to inhibit disulfide bond formation. Diethyl maleate depletes GSH in exposed cells and facilitates disulfide bond formation within the cells. TCEP facilitates a faster S–S reduction process, thereby decreasing disulfide bond formation within the cells. Because the cell survival rate was <10% after culturing the cells in the low-glucose medium for 24 h, any potential drug-induced enhancement of cell death would not be reflected in the cell survival rate. Therefore, we designed the time of low-glucose medium treatment (3, 6, 12, and 24 h) to assess the cell survival rate and chose 12 h as the optimal treatment duration. We included diethyl maleate and TCEP in the low-glucose medium and assessed the cell survival rate after culturing the cells for 12 h under different treatment conditions. The results revealed that diethyl maleate exhibited a promoting effect on the cell death mechanism, while TCEP exhibited a protective effect. The findings demonstrated that the cell death mechanism triggered by glucose starvation in SLC7A11^high^-expressing OC cells is associated with the formation of disulfide bonds in the cell, which facilitates this cell death mechanism.

Glucose starvation is one of the main forms of metabolic stress in cancer cells, impairing glycolysis and PPP. This deficiency induces oxidative stress, elevates the production of ROS, and compromises the antioxidant system, leading to oxidation–reduction imbalance and cell death (Bray et al., 2018). Do the cell death mechanisms induced in OC cells in glucose starvation conditions correlate with increased ROS and oxidative stress in the cell? We used ROS detection kits based on ROS-CAT to measure the ROS levels in OVCAR 3 and IOSE 80 cells before and after glucose starvation. The results revealed that the ROS level in OVCAR 3 cells increased significantly under glucose starvation conditions; however, no significant change was observed in the ROS level of IOSE 80 cells. Therefore, we hypothesized that the increase in the ROS level in OVCAR 3 cells under glucose starvation conditions results in oxidative stress and cell death. Tempol and NAC have ROS-scavenging effects; however, Tempol functions as a superoxide dismutase (SOD) that directly neutralizes free radicals to mitigate ROS (Wilcox, 2010), while NAC primarily inhibits ROS by supplying cellular cysteine and facilitating GSH synthesis. Additionally, NAC may prevent the accumulation of cysteine or other disulfides under glucose starvation conditions by disulfide exchange (Cys–Cys + NAC → Cys + NAC–Cys) (Aldini et al., 2018; Pedre et al., 2021). After culturing OVCAR 3 and IOSE 80 cells in the low-glucose medium for 24 h and adding Tempol or NAC, we assessed cell viability. The results revealed that Tempol was unable to prevent OVCAR 3 cell death, indicating that the cell death induced by glucose starvation in OVCAR 3 cells is unrelated to the increase in the intracellular ROS level. NAC can prevent this type of death. Since NAC can supply intracellular cysteine and enhance GSH synthesis to eliminate ROS, we propose that increasing the intracellular cysteine levels and diminishing intracellular disulfide bond formation can mitigate glucose starvation-induced OC cell death.

Previous studies have demonstrated that the cell death mechanism in OC cells during glucose starvation conditions is associated with increased SLC7A11 expression. Does NAC reduce the levels by inhibiting SLC7A11 expression? To answer the question, we observed the difference in the SLC7A11 expression level in OVCAR 3 cells before and after NAC treatment. The results revealed no significant difference in SLC7A11 levels in OVCAR 3 cells before and after NAC treatment. This proves that the rescue effect of NAC is not accomplished by inhibiting SLC7A11 expression.

In glucose-rich conditions, high SLC7A11 expression in OC cells facilitates the transport of extracellular cysteine into the cell, where it is utilized to generate NADPH through PPP. Cysteine, along with glutamate and glycine, forms the tripeptide glutathione (GSH), which regulates the oxidation–reduction level of the cell. However, in glucose starvation conditions, due to the lack of sugar metabolic raw materials, glycolysis and the PPP are inhibited. The PPP is the primary source of NADPH for the cell; therefore, NADPH generation is reduced. However, high SLC7A11 expression in OC cells, coupled with glucose starvation that further induces SLC7A11, results in continuous transport of cysteine from the extracellular space into the cell, consuming intracellular NADPH and leading to cysteine accumulation and disulfide stress, which causes cell death.

Our research has revealed that the mode of cell death in ovarian cancer cells under glucose starvation is closely related to SLC7A11 expression. In addition, under glucose starvation conditions, the NADPH in ovarian cancer cells decreased and disulfides accumulated, which is closely related to ovarian cancer cell death. Previous studies by Liu et al. (2023) have indicated that actin cytoskeleton proteins are particularly susceptible to the influence of disulfide bond stress caused by excessive accumulation of disulfide bond molecules within cells. If abnormal disulfide bonds between actin and cytoskeletal proteins are not repaired, it will lead to actin network collapse and cell death. Therefore, we also reasonably speculate that ovarian cancer cell-death under glucose starvation conditions is also caused by the collapse of the actin skeleton protein network. Future studies should include enhanced observation of actin cytoskeletal dynamics and further investigate the underlying mechanisms of cell death.

In glucose-starved conditions, SLC7A11^high^ OC cells undergo cell death, and this type of cell death is associated with intracellular NADPH depletion and disulfide compound accumulation. Therefore, we consider the possibility of disulfide death occurring in SLC7A11^high^ OC cells in glucose-starved conditions.

This study has some limitations. First, only OVCAR 3 OC cells were used. In addition, it lacks *in vivo* data. If we had included more OC cells, performed animal experiments, and provided clinical samples for validation, our evidence would have been more convincing. Accordingly, in our subsequent studies, we will conduct additional experiments to further verify and investigate the underlying mechanisms.

Due to the lack of an *in vivo* study, future studies may validate these findings in patient-derived xenograft models (Kang et al., 2024; Lee et al., 2024). On the other hand, recent studies have made new advancements in cancer diagnostics and monitoring, such as liquid biopsies (Jahangiri, 2024), molecular barcode detection systems (Ohyama et al., 2024), and methylation analysis (Gonzalez et al., 2024). The latest advancements in liquid biopsy technology have provided groundbreaking tools for cancer diagnosis and treatment monitoring. Research indicates that circulating tumor DNA (ctDNA) can be utilized to monitor treatment response and recurrence in patients with neuroblastoma in real time (Jahangiri, 2024). The SLC7A11-mediated disulfidptosis mechanism identified in our study may also be detectable and trackable through liquid biopsy techniques. Emerging molecular barcoding sequencing technologies have significantly enhanced the sensitivity and specificity of ctDNA detection, enabling the identification of low-frequency mutations (Ohyama et al., 2024). Additionally, methylation analysis—an integral component of liquid biopsy—has demonstrated efficacy in identifying early-stage biomarkers for cancers such as breast cancer (Gonzalez et al., 2024). This non-invasive approach holds promise for monitoring epigenetic alterations linked to SLC7A11 in ovarian cancer patients. Our subsequent research may apply this new detection technology to SLC7A11 gene analysis in solid ovarian tumors.

## Conclusion

Glucose starvation induces high SLC7A11 expression in OC cells, leading to cell death. This type of cell death is associated with NADPH depletion in the cell and disulfide accumulation. Consequently, it is considered that double sulfide death may occur in SLC7A11^high^ OC cells under glucose starvation conditions.

## Data Availability

The original contributions presented in the study are included in the article/Supplementary Material, further inquiries can be directed to the corresponding author.

## Abbreviations

OC, ovarian cancer; RCD, regulated cell death; xCT, the glutamate/cystine antiporter solute carrier family 7 member 11; PET, positron emission tomography; CT, computed tomography; FDG, 18-fluorodeoxyglucose; TCA, tricarboxylic acid cycle; WB, Western blot; PCR, polymerase chain reaction; qRT-PCR, real-time quantitative polymerase; ROS, reactive oxygen species; SAS, sulfasalazine; NAC, N-acetylcysteine; NADP^+^, nicotinamide adenine dinucleotide phosphate; NADPH, nicotinamide adenine dinucleotide phosphate; GSSG, oxidized glutathione disulfide; GSH, reduced glutathione; TCEP, tris(2-carboxyethyl)phosphine; PPP, pentose phosphate pathway; DMSO, dimethyl sulfoxide; αKG, α-ketoglutaric acid.
