# Supplementary figures and images for: The effect of disulfidptosis induced by glucose starvation in SLC7A11high ovarian cancer cells
Source: Front Cell Dev Biol. 2025 Dec 8;13:1608150. doi: 10.3389/fcell.2025.1608150 (PMC12719518; doi:10.3389/fcell.2025.1608150)

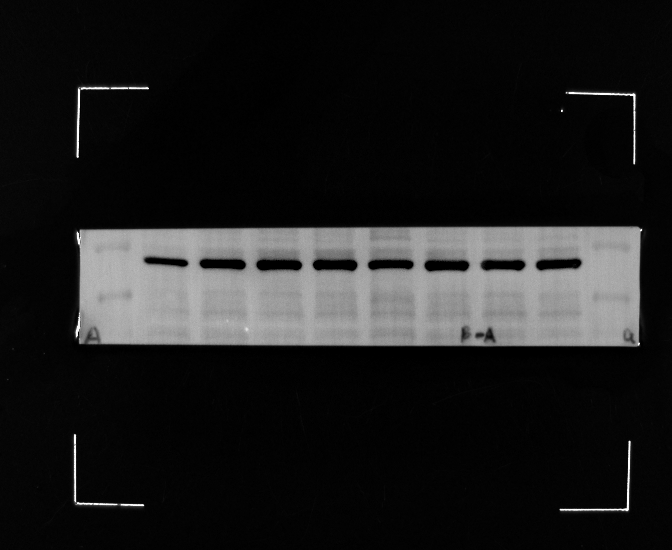

Supplement: Supplementary file 1 [file DataSheet1.zip › WB/Actin.tif]

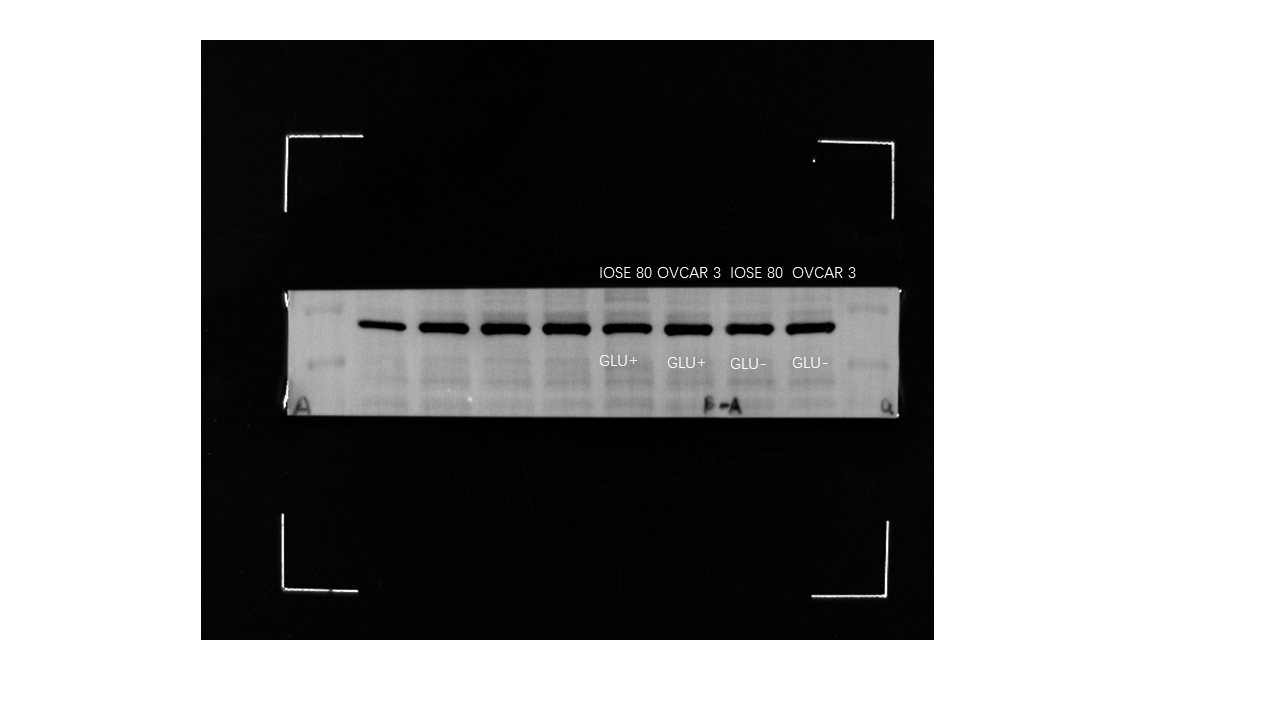

Supplement: Supplementary file 1 [file DataSheet1.zip › WB/figure legends/Actin.TIF]

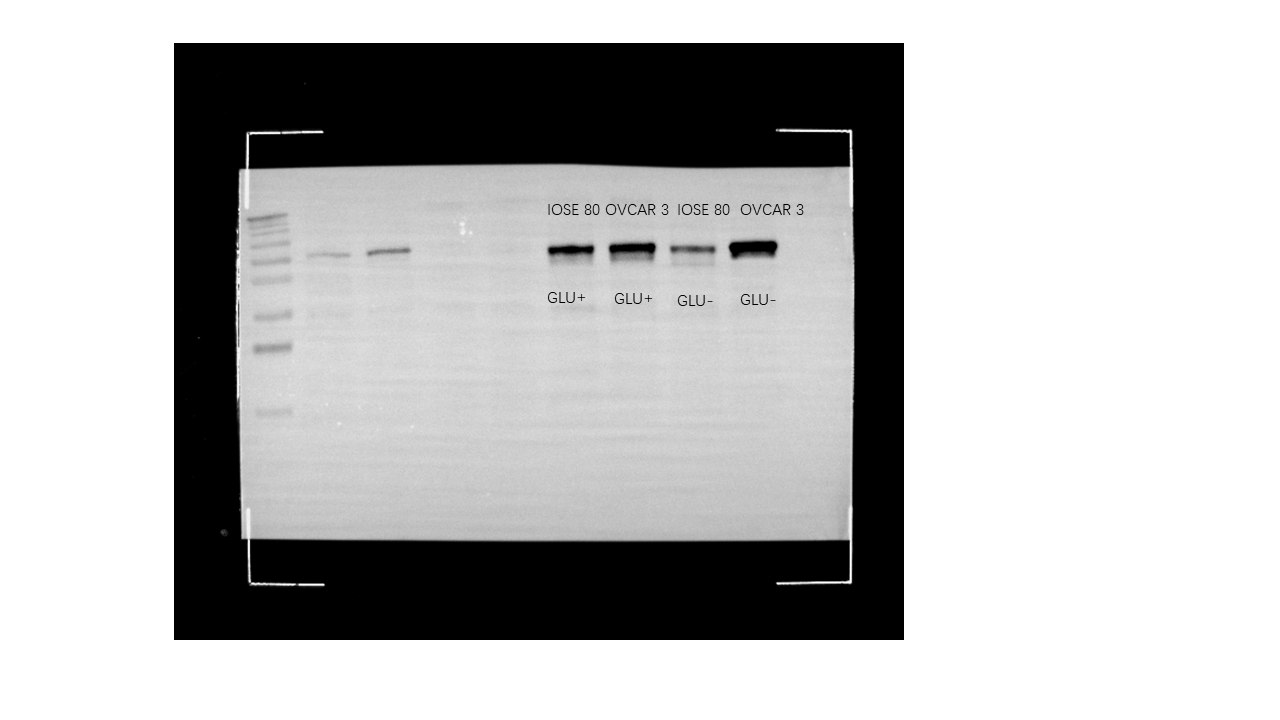

Supplement: Supplementary file 1 [file DataSheet1.zip › WB/figure legends/xCT.TIF]

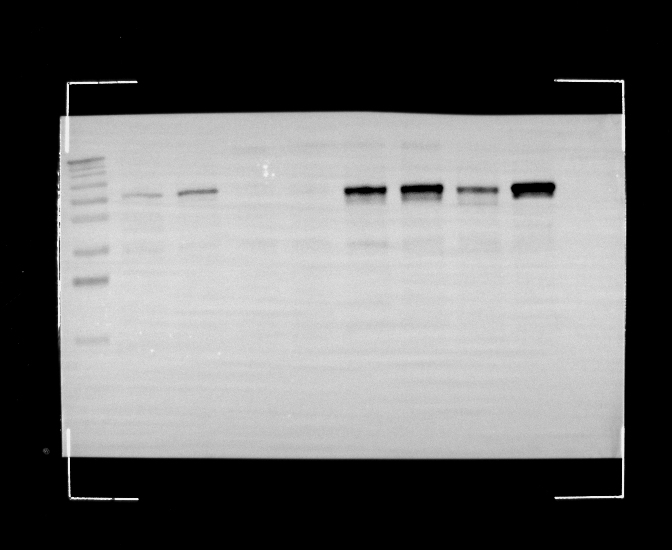

Supplement: Supplementary file 1 [file DataSheet1.zip › WB/xCT.tif]
